# The Flavor Profiles of Highland Barley Fermented with Different Mushroom Mycelium

**DOI:** 10.3390/foods11243949

**Published:** 2022-12-07

**Authors:** Kai Wang, Cuicui Yang, Ziyan Dai, Zhenxiang Wen, Yin Liu, Xi Feng, Ying Liu, Wen Huang

**Affiliations:** 1 College of Food Science and Technology, Huazhong Agricultural University, Wuhan 430070, China; 2 Quality and Safety Inspection Center for Agricultural and Livestock Products, Haidong 810699, China; 3 Wuhan Huanghelou Essence and Flavor Co., Ltd., Wuhan 430040, China; 4 Department of Nutrition, Food Science and Packaging, San Jose State University, San Jose, CA 95192, USA

**Keywords:** edible mushrooms, E-nose, sensory evaluation, GC-MS, volatiles

## Abstract

Highland barley was fermented with *Cordyceps militaris*, *Stropharia rugoso-annulata*, *Morchella esculenta*, *Schizophyllum commune* and *Tremella sanguinea*. The flavor profiles were investigated by electronic nose (E-nose), headspace solid-phase microextraction gas chromatography-mass spectrometry (HS-SPME-GC-MS) and sensory evaluation by train panel. Fermentation with mushroom mycelium was able to change the aroma profile of highland barley. The original strong grassy taste was reduced due to a decrease in hexanal, decanal and 2-pentylfuran, and new aromatic flavors (floral, sweet and mushroom fragrance) were acquired after fermentation. The overall flavor of the fermented highland barley varied with mushroom strains. *Schizophyllum commune* gave a heavier sour taste to the fermented highland barley. However, fermentation with *T. sanguinea* increased the content of methyl 4-methoxybenzoate making the sample difficult to accepted. Fermentation with *C. militaris*, *M. esculenta*, and *S. rugoso-annulata* increased the volatile contents. The high levels of 1-octen-3-ol and esters gave a strong mushroom, oily and fruity flavor. *Morchella esculenta* showed the best performance and the highest acceptance in the fermented highland barley. Our results suggest that fermentation with mushroom mycelium can improve the flavor of highland barley, which provides an innovative utilization of highland barley.

## 1. Introduction

Highland barley (*Hordeum vulgare* L. *var. nudum Hook.f.*) grows at high altitude and in cold regions. It is mainly distributed in Tibet, Qinghai and other places in China and is an important highland cereal crop. It is resistant to barrenness and cold, but with a high yield and wide adaptability [1]. Highland barley has a high protein (10–17%), dietary fiber (11%–34%) and vitamin (1.5–2.5%) content, but with low fat (2–3%) and carbohydrate (65–68%) contents [2]. The “three high and two low” nutritional characteristics of highland barley give it potential to be an ingredient in healthy foods [3]. Currently, highland barley mainly has been used as a main ingredient in noodles, cookies and beverages [4]. However, due to its grainy texture and poor processing properties, the application of highland barley in foods is still limited. Compared with other grains, the high levels of volatile compounds, such as hexanal and decanal, provide a strong grassy flavor in highland barley [5]. As a result, consumers’ acceptance of highland barley products is low [6]. Therefore, it is necessary to find appropriate processing methods to modify highland barley’s sensory characteristics and broaden its application.

Currently, grain processing property modification can be achieved by physical, chemical and biological methods [7]. Physical modifications are generally divided into thermal and non-thermal modification. Thermal modification includes pre-paste and hydrothermal treatments (heat and moisture treatment (HMT) and annealing (ANN)) [8,9], while non-thermal modifications include high-pressure treatment (HPP), micronization, ultrasonic, pulsed electric field (PEF), etc. [10,11]. Chemical methods use derivatization reactions (etherification, esterification, cross-linking) or hydrolysis and oxidation reactions to modify grain chemical structures [12]. Compared with other modification methods, fermentation has the advantages of lower cost and high yield. Beneficial metabolites can be produced during fermentation as well [13]. Large molecules are degraded to small molecules after fermentation, which improves flavor characteristics [14]. Strains commonly used in the food industry are *Lactobacillus plantarum* and yeast. They can provide strong sour and winey flavors to fermented foods, respectively [15].

Mushroom mycelium is easy to grow and has aromatic flavors during its growth and development. Solid fermentation of grains with mushroom mycelium brings out the sweetness of flowers and herbs [16]. It has been reported that the fermentation of soybean residues with *Stenotrophomonas* edible fungi was able to reduce the legume flavor, and the flavor profile of the fermented soybean residue were described as floral and sweet [15].The fermentation of wheat bran using *Fomitopsis pinicola* resulted in decreases in aldehyde and lipid contents and an increase in ketones and phenols [17]. It has also been found that fermentation of bran with mushroom mycelium can improve its nutritional values and form aromatic components [18]. This evidence provides the basis for the fermentation of mushroom mycelium to improve the flavor of grains. However, there is limited research on the flavor modification of highland barley with mushroom mycelium.

Differences in metabolism among mushroom strains could lead to different flavor profiles in the final product [19]. Therefore, strain selection is considered to be a key step. Screening the suitability of mushroom strains would be of great importance. The aims of this study were to modify highland barley with a solid-state fermentation by using five different edible mushroom strains (*C. militaris*, *S. rugoso-annulata*, *S. commune*, *T. sanguinea* and *M. esculenta*). The aroma characteristics of different types of fermented highland barley were investigated. The volatile profiles of fermented highland barley were analyzed by E-nose and headspace solid-phase microextraction (HS-SPME) with GC-MS. A sensory flavor evaluation was also used to analyze the aroma profiles.

## 2. Materials and Methods

### 2.1. Material

Highland barley (species: *Belly Yellow*) was purchased from Qinghai Xinning Biotechnology Co., Ltd. (Xining, Qinghai, China) and stored at room temperature. Five mushroom strains (*C. militaris*, *S. rugoso-annulata*, *S. commune*, *T. sanguinea* and *M. esculenta*) were provided by the Institute of Applied Fungal Research, Huazhong Agricultural University (Wuhan, China). The domesticated strains were collected from the field and stored in PDA slant medium at 4 °C. Potato dextrose agar (PDA) medium was obtained from Sinopharm Chemical Reagent Co. The n-alkane standards (C5–C24) were purchased from Sigma Chemical Company (St. Louis, MO, USA).

### 2.2. Sample Preparation

The original slant mycelia of the five strains were cultured on PDA, then incubated in a biochemical incubator (Wuhan Ruihua Instrument Equipment Co., Ltd., Wuhan, China) for 6 days at 25 °C. The mycelium was activated by inoculating it on a different petri dish. After that, the activated strains were cultured in liquid shaking flasks. Clean triangular 250 mL flasks were prepared and filled with 100 mL of prepared liquid fermentation medium, then autoclaved at 121 °C for 30 min in a pressure steam sterilizer (Shanghai Sanshen Medical Devices Co., Ltd., Shanghai, China) and cooled for use.

Each of 250 g highland barley sample was soaked in distilled water for 3 h and then was added to a 1000 mL mason jar. The mason jars were then autoclaved at 121 °C for 30 min and cooled to room temperature. The liquid strain was inserted at 5% of the dry weight of highland barley, followed by fermentation at 25 °C for 12 days [16]. After that, the fermented samples were dried in an oven at 50 °C to reach a constant weight (moisture content was 11.7%). The five fermented samples were named CM-HB (*C. militaris*), SR-HB (*S. rugoso-annulata*), ME-HB (*M. esculenta*), SC-HB (*S. commune*) and TS-HB (*T. sanguinea*). Finally, the fermented samples were ground into 40-mesh powder and stored at 25 °C for further study.

### 2.3. E-Nose Analysis

A FOX-4000 electronic nose system from Alpha M.O.S. (Toulouse, France) was used for electronic nose analysis. The instrument consists of 16 metal oxide sensors combined with a headspace autosampler HS100. A total of 2 g of the sample was added to a 10 mL vial, covered with a Teflon rubber cap, and equilibrated for 120 s at 50 °C with stirring (500 rpm). Dry air was used as the carrier gas at a flow rate of 150 mL/min. An equilibrated top space (2500 μL) was injected into the e-nose via a 2500 μL gas-tight syringe (60 °C) at a rate of 2500 μL/s. The acquisition time and delay time between successive injections were set to 120 s and 300 s, respectively [20].

### 2.4. SPME-GC-MS Analysis of Volatile Flavor Compounds

An SPME (solid phase microextraction) autosampler equipped with 50/30 μm divinylbenzene/carboxyl/polydimethylsiloxane (DVB/CAR/PDMS) fibers (Supelco, Bellfonte, PA, USA) was used to extract volatile compounds from the samples. The homogenate of highland barley and post-fermented highland barley powder (1 g of sample powder in 10 mL of sodium chloride saturated solution) was added to a 20 mL vial equipped with a magnetic stirring bar. The vial was immediately sealed with a PTFE septum (Supelco, Bellfonte, PA, USA). The samples were equilibrated at 60 °C for 10 min and then the fibers were inserted into the vial for 40 min to extract the volatile compounds. Finally, the fibers were inserted into the inlet of the GC and desorbed in splitless mode for 5 min.

Agilent (7890B-7000D) was used for the analysis of volatile compounds. An HP-5MS non-polar capillary column (30 m × 0.25 mm inner diameter, 0.25 mm film thickness, Agilent Technologies, Santa Clara, CA, USA) was installed at the GC. GC conditions were set as follows: helium carrier gas at a flow rate of 1 mL/min; injector temperature of 250 °C; oven temperature initially programmed to hold at 50 °C for 2 min, then 3 °C/min to 90 °C for 5 min and finally 10 °C/min to 260 °C for 1 min. The ionization source temperature was set to 230 °C. MS was obtained using the electron impact mode at 70 eV in the range of 50 *m*/*z* to 450 *m*/*z* [21]. Qualitative analysis of the volatile components of unknown compounds in the samples was obtained by computer search of NIST 17.0 and demonstration of a library of standard mass spectra. Only compounds with matches ≥80% were searched and recorded. Volatile compounds were identified by comparing the Kovats retention index (RI) and MS fragmentation patterns with mass spectra from the NIST17 library. RI values were calculated for all compounds studied using a series of n-alkanes (C5–C24) sampled under the same chromatographic conditions. Only compounds with matching RI values and MS spectra were reported here. Quantification was calculated by normalizing peak areas based on relative percentage content. Corrosion of SPME fibers and capillary columns and unidentified peaks were removed. Only the identified peaks were used for normalization.

### 2.5. Analysis of Relative Odor Activity Value

Based on the content of volatile compounds in the six samples before and after fermentation obtained by GC-MS analysis, the key volatile compounds in the six samples were identified using the ROAV method. The component that contributed most to the odor of the sample was defined as ROAV_stan_ and given a value of 100, and the other volatile components were calculated as follows [22]

(1) $$ROAV_{\begin{array}{l} i \end{array}}\approx \frac{C_{ri}}{C_{stan}}\times \frac{T_{stan}}{T_{i}}\times 100$$

where *C_ri_* and *T_i_* are the relative content (peak intensity of each compound as a percentage of the total peak intensity of the compounds examined for determination) and the sensory threshold for each volatile component, respectively. *C_stan_* and *T_stan_* are the relative levels and thresholds, respectively, of the compounds that contribute the most to the main odor of the sample.

### 2.6. Sensory Evaluation

Ten students majoring in foods (5 men and 5 women) were selected to conduct the sensory evaluations [23]. All team members gained extensive experience in sensory characterization of various food samples and built a database of sensory descriptions based on their long experience. Prior to the analysis, the sensory analysis team attended four training sessions (spending 2 h each) to enhance the sensory description of the fermented products until they had all gained enough experience in sensory analysis and were quite familiar with sensory evaluation. Seven flavor profiles were developed through group discussions, which ultimately identified grassy aroma (20 μg·kg^−1^ hexanal), acidity (sour odor from fermentation), earthy, oily (fatty odor), mushroom (5 μg·kg^−1^ 1-Octen-3-ol), sweet (fruit clear sweet 150 μg·kg^−1^ hexyl acetate) and spicy (peppery spice pungency). The sensory evaluation was based on a ten-point system, from 0–10 points, with a gradual increase in aroma intensity, with 0 points indicating no aroma intensity, 5 points indicating medium aroma intensity and 10 points indicating very strong aroma intensity. Each sample was evaluated three times at an interval of 10 min at room temperature. The average score of each aroma attribute and overall acceptance was taken as the final evaluation result.

### 2.7. Data Processing

Principal component analysis (PCA) and radar fingerprint analysis were performed using AlphasoftV9.1 software (Alpha MOS Co., Toulouse, France).

One-way ANOVA (Duncan’s multiple range test) was used for data analysis with SPSS 22.0 software (demo version, Armonk, NY, USA). *p* < 0.05 was considered a significant difference. Cluster heat map analysis was performed using Origin 2022 software (Origin Lab Corporation, Northampton, MA, USA). All experiments were repeated three times.

The Scientific Ethics Committee of Huazhong Agricultural University approved the study (ID Number: 202210310001).

## 3. Results and Discussion

### 3.1. Electronic Nose Analysis of Highland Barley

**Figure 1:**
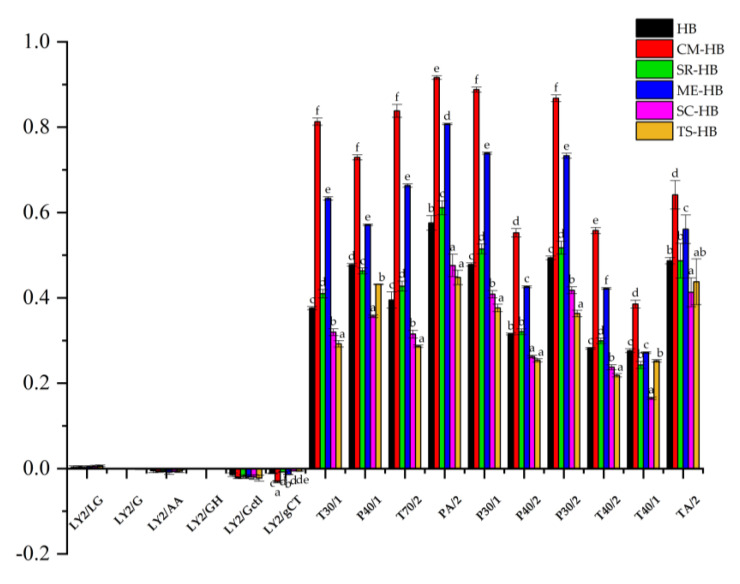
E-nose response value plot for original and fermented highland barley samples: HB (original highland barley), CM-HB (fermented highland barley with *C. militaris*), SR-HB (fermented highland barley with *S. rugoso-annulata*), ME-HB (fermented highland barley with *M. esculenta*), SC-HB (fermented highland barley with *S. commune*), TS-HB (fermented highland barley with *T. sanguinea*). Different superscript lowercase letters in a row (a–f) represent statistically significant differences between the mean values at *p* < 0.05 determined by one-way ANOVA.

E-nose is a non-destructive, comprehensive and rapid method to assess food quality [24]. As shown in Figure 1, the sensor response values of the six groups of samples (HB, CM-HB, SR-HB, ME-HB, SC-HB and TS-HB) were significantly different (*p* < 0.05). This indicated that the aroma of the samples changed significantly after the fermentation of highland barley with mushroom mycelium.

The response values (0~0.2) of the first six sensors (LY2/LG, LY2/G, LY2/AA, LY2/GH, LY2/Gctl and LY2/gCT) were significantly lower than those of the other sensors (Figure 1). The main differences between samples were in the other ten sensors (T30/1, P40/1, T70/2, PA/2, P30/1, P40/2, P30/2, T40/2, T40/1 and TA/2). All the samples had high response values with differences of 0.2 to 0.85, which was attributed to natural gases, fermented flavors, oxidized gases and aromatic substances in the foods.

Figure 1 shows the significant differences among the samples in terms of sensor response values. Both CM-HB and ME-HB had higher response values in T30/1, P40/1, T70/2, PA/2, P30/1, P40/2, P30/2 and T40/2 sensors compared to other samples. The response values of SR-HB were similar in profile to the HB sensor response values. In contrast, the SC-HB and TS-HB samples had low response values compared to those in the ten sensors.

**Figure 2:**
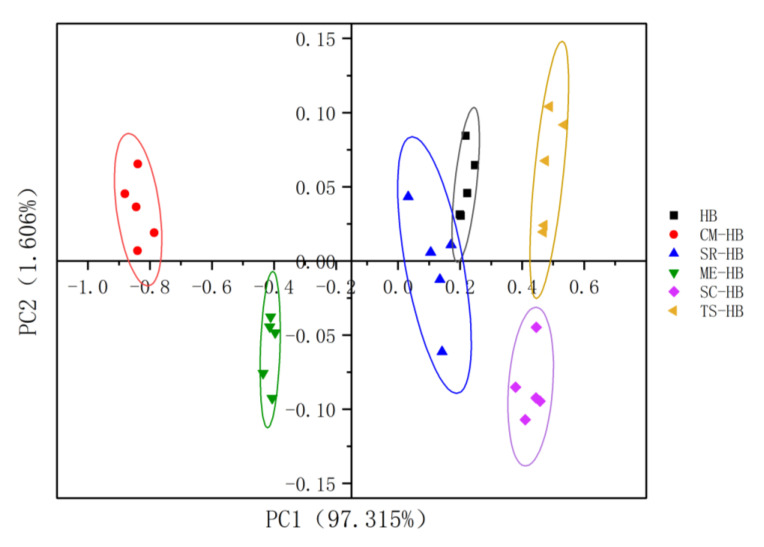
PCA of E-nose analysis for original and fermented highland barley samples: HB (original highland barley), CM-HB (fermented highland barley with *C. militaris*), SR-HB (fermented highland barley with *S. rugoso-annulata*), ME-HB (fermented highland barley with *M. esculenta*), SC-HB (fermented highland barley with *S. commune*), TS-HB (fermented highland barley with *T. sanguinea*).

PCA is a statistical tool to explain the differences between samples by their principal components. In general, the feasibility of the method can be considered when the total contribution rate exceeded 85% [25]. As shown in Figure 2 the contribution of PC1 was 97.32% and of PC2, 1.61%, with a cumulative contribution of 98.921%, indicating that the electronic nose analysis could represent most of the volatile flavor information of the different samples. The results showed clustering of samples in the PCA plot [26]. There was partial overlap between SR-HB and HB. SC-HB and TS-HB were located on the right side of the X-axis. However, ME-HB and CM-HB were located on the left side of the X-axis. The results indicate that the volatile odorants varied significantly among all the samples fermented with different edible mushrooms.

### 3.2. Volatile Compound Analysis of Highland Barley

Information on volatile metabolites in different types of fermented highland barley samples is shown in Table 1. A total of 58 volatile flavor compounds were detected in the six samples, including 16 aldehydes, 8 alcohols,7 ketones, 9 esters, 5 acids and 13 other compounds. As shown in Table 2, 18 volatile substances were detected in highland barley including 7 aldehydes, 1 alcohol, 3 esters, 2 ketones and 5 other substances. After fermentation, the amounts of substances detected in the products (SC-HB, TS-HB, SR-HB, ME-HB and CM-HB) were 24, 22, 23, 17, and 27, respectively. Among them, differences were produced in the type and amount of volatile components depending on the fermentation strain. The results also indicated that different types of mushroom mycelium could lead to varied levels of volatile-flavor-compound formation in the fermented highland barley [27].

**Figure 3:**
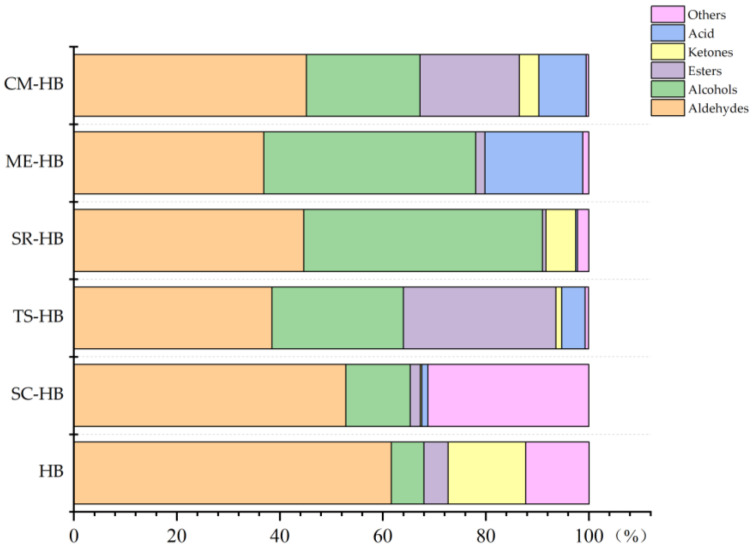
Changes in the total amount and types of volatile compounds in original and fermented highland barley samples: HB (original highland barley), CM-HB (fermented highland barley with *C. militaris*), SR-HB (fermented highland barley with *S. rugoso-annulata*), ME-HB (fermented highland barley with *M. esculenta*), SC-HB (fermented highland barley with *S. commune*), TS-HB (fermented highland barley with *T. sanguinea*).

Aldehydes are mainly produced by lipid oxidation, thermal degradation, microbiological reaction and Maillard reaction. Because of their lower threshold, they had a greater impact on the final flavor profiles of products. In addition, aldehydes have strong flavor effects that overlap with those of many other substances [28]. As shown in Figure 3, the dominant volatiles in the six samples were aldehydes. In the unfermented highland barley, the relative content of aldehydes was also high. Among them, decanal, hexanal and heptanal showed grassy smell, flowery and fruity flavors, respectively [29]. However, the content of decanal, hexanal and heptanal in the products after fermentation of the five mushroom mycelium species was significantly reduced, indicating that the original characteristics of the off-flavors produced were significantly reduced after fermentation. Compared with the original highland barley, eight new aldehydes were detected in the fermented samples. In addition, benzaldehyde and phenylacetaldehyde were detected in all samples, and their contents increased after fermentation. They were derived from the decarboxylation of phenylalanine catalyzed by aromatic l-amino acid decarboxylase. Benzaldehyde had a special cherry and almond aroma, while phenylacetaldehyde had the sweetness of rose and honey to give the highland barley a richer odor expression. After analyzing the changes in the types and contents of the aldehydes of the products before and after fermentation, it was found that the relative contents of decanal, hexanal and heptanal present in the original plateau barley decreased after fermentation, indicating a decrease in grassy, floral and fruity aromas. In contrast, phenylacetaldehyde, benzaldehyde and other newly produced aldehydes brought fruit and floral aromas. Among them, the relatively high contents of trans-2-Octen-1-al, gamma-nonanoiclactone, 2-Ethyl-2-hexenal, (E)-2-butyloct-2-enal and cyclohexanecarboxaldehyde present in the CM-HB sample brought strong fatty and fruity aromas.

Alcohols produced by fermentation and lipid oxidation had significant contributions to flavor [30]. As shown in Figure 3, the number and amount of alcohols increased after fermentation. In particular, 1-Octen-3-ol increased significantly after fermentation. 1-Octen-3-ol has a signature mushroom odor, earthy flavor and rose odor [31]. 2-Phenylethanol was detected in the samples of SC-HB, TS-HB, ME-HB and CM-HB, which provided aromas similar to honey and rose. Linalool and nerolidol were also detected in CM-HB, TS-HB and SR-HB samples, and CM-HB had the highest levels. The results indicated that fermentation brought a sweet smell to the product, which might be due to microbiological reactions and enzymatic activities [32].

Esters produced through esterification can give foods a sweet aroma and oily taste [33]. As shown in Figure 3, the number and amount of esters in highland barley after fermentation were different. After fermentation, ester levels in TS-HB and CM-HB increased, and the CM-HB samples were most rich in ester species. Ethyl palmitate, ethyl linoleate, ethyl oleate, ethyl myristate and ethyl stearate, which were not found in original highland barley, were detected in CM-HB. These volatile compounds could add floral and fruity notes. Esters amounts in SC-HB, ME-HB and SR-HB were decreased, whereas their diversity was increased. However, low levels of esters can also add positive tastes to foods [34].

Ketones can be formed from amino acids’ thermal degradation or the oxidation of polyunsaturated fatty acids [35]. The contents of ketones in fermented samples were decreased, which indicated that ketones contributed little to the odor of the fermented products [36]. However, acids are mainly produced from the decomposition and enzymatic hydrolysis of fat, or as metabolites during edible mushroom fermentation. In this study, acids were only detected in the fermented samples. Among them, ME-HB showed the highest level of saturated fatty acids. The fatty acids have high odor thresholds and little effect on the overall odor. The other volatile compounds in the highland barley samples were mainly hydrocarbons and 2-pentylfuran. The high content of 2-pentylfuran contributed a green, bean and fruity smell to highland barley. After fermentation, the original hydrocarbons and furans were greatly decreased or disappeared. Some phenolic compounds were detected in the fermented samples. These phenolic compounds are mainly produced by decarboxylation of phenolic carboxylic acids and thermal degradation of cellulose, lignin or hemicellulose [37].

In summary, fermentation with mushroom mycelium could increase the richness of volatile compounds and improve the aroma of highland barley. The original grassy taste and astringency of highland barley disappeared after fermentation due to decreases in decanal, hexanal and heptanal. The increase in phenylacetaldehyde, benzaldehyde and 1-octen-3-ol brought floral, sweet and mushroom aromas to the fermented highland barley. In particular, the higher content of the newly produced substance furan-3-carboxaldehyde detected in the SC-HB sample can provide a burnt odor. Producing 3-(methylthio)propionaldehyde brought an irritating sour odor. These newly produced volatile flavor substances were partly metabolites of the raw material in the highland barley when it was fermented with the mushroom mycelium and partly from the raw material.

A recent study carried out analysis of volatile compounds of four mushrooms (*Agaricus bisporus* ssp. *bisporus*, *Agaricus bisporus* ssp. *brunnescens*, *Lentinula edodes* and *Grifola frondose*) using GCMS. The 3-octanone, 1-hexanol and 1-octen-3-ol contained in the measured samples were found to be the secondary metabolites present in most of the mushrooms. These compounds were detected in samples from different mushrooms after mycelial fermentation [38]. Some researchers also found that 3-(methylthio)propionaldehyde was the most predominant aromatic active compound in Boletus edulis [39]. This compound was detected in SC-HB samples and had a significant impact on the overall flavor of the product. In a study on the use of edible fermentation to improve food flavor, researchers used four edible bacteria to ferment soybeans to remove off-flavors. It was found that substances such as hexanal, which brings off-flavors to soybeans, were consumed and utilized in the growth of mycelium [15]. Moreover, substances that present floral aromas such as phenylethanol, nonanal and linalool were detected in the fermented products, which is consistent with the results of this study.

### 3.3. Thermogram Analysis of Volatile Compounds

**Figure 4:**
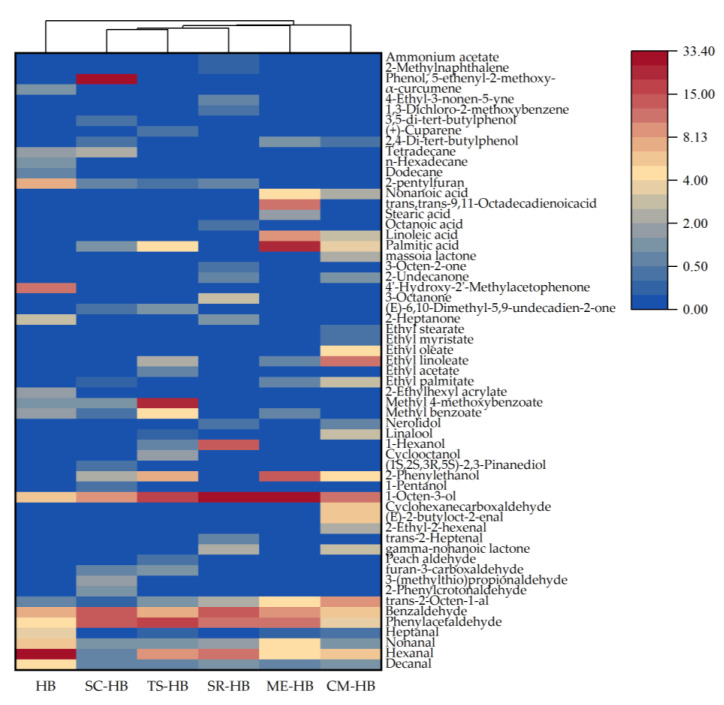
Thermogram analysis of volatile flavor compounds for original and fermented highland barley samples: HB (original highland barley), CM-HB (fermented highland barley with *C. militaris*), SR-HB (fermented highland barley with *S. rugoso-annulata*), ME-HB (fermented highland barley with *M. esculenta*), SC-HB (fermented highland barley with *S. commune*), TS-HB (fermented highland barley with *T. sanguinea*).

The heatmap based on the type and amount of volatile compounds is shown in Figure 4. The differences between various volatile compound levels are represented by different shades of color, which enable a more visual display of differences between samples [38]. It is relatively clear from the thermogram that fermentation with mushroom mycelium increases the abundance of volatile compounds and improves the aroma of highland barley. After fermentation, the original grassy and astringent taste of highland barley disappeared due to the reduction in decanal, hexanal and heptanal. The increase in phenylacetaldehyde, benzaldehyde and 1-octen-3-ol brought floral, sweet and mushroom aromas to the fermented highland barley. It is obvious from Figure 4 that among the volatile compounds in CM-HB samples, esters, including methyl benzoate (Honey, Flowers), ethyl palmitate (Fragrance, Milk), ethyl linoleate (Fatty), ethyl oleate (Cocoa), ethyl myristate (Iris aroma, Fat), andethyl stearate (Slightly waxy, Irritating), and some alcohols such as linalool (Floral, Lily of the valley) and nerolidol (Green, Floral, Fruity aromas) are relatively high in content. ME-HB contains some ethyl linoleate, methyl benzoate and ethyl palmitate with an overall floral, sweet and oily aroma, but also has a relatively high content of unsaturated fatty acids. The relatively high content of 3-(methylthio)propionaldehyde (Stench) in SC-HB and the high content of methyl 4-methoxybenzoate (Peppers, Herbs, Dried fruits) in TS-HB had a negative effect on the overall flavor presentation. Meanwhile, the SR-HB sample was richest in aroma composition among the six samples.

### 3.4. Relative Odor Activity Value (ROAV) Analysis of Volatile Compounds

The ROAV method is used to determine the contribution of each volatile compound to the primary odor. The greater the ROAV value, the more the contribution to the primary odor. Compounds with ROAV > 1 are key volatile compounds, and compounds with 0.1 ≤ ROAV < 1 have important influences on the overall flavor [40]. Before fermentation, the odor of highland barley was mainly from decanal, hexanal, nonanal, heptanal, phenylacetaldehyde and 2-pentylfuran (Table 3). These compounds made the highland barley exhibit grassy, floral and leguminous odors. After fermentation, the contents of these components decreased. All the fermented samples showed a high level of 1-octen-3-ol, which has a mushroomy odor.

High levels of 2-phenylethanol were detected in SC-HB, TS-HB, ME-HB and CM-HB samples, which gave a floral aroma. In SC-HB, the presence of 3-(methylthio) propanal with low threshold and high content brought an unpleasant taste. Ethyl benzoate and ethyl acetate in TS-HB played an important role in the aroma with floral notes. However, the presence of methyl anthranilate also brought unacceptable spicy notes. The high content of phenylacetaldehyde in both samples produced a bitter almond flavor that was not liked by consumers. For the SR-HB, ME-HB and CM-HB samples, decanal, hexanal, nonanal, phenylethylaldehyde, trans-2-octen-1-aldehyde and 1-octen-3-ol played a key role in the overall flavor. Other minor aldehydes, esters and ketones (benzaldehyde, 1-hexanol, ethyl myristate, etc.) mostly had a minor contribution. For example, linalool and 2-undecanone were present in the CM-HB samples, which provided floral and citrus aromas to the sample. It can be seen that in highland barley fermented with *C. militaris*, *S. rugoso-annulata* or *M. esculenta*, the flavor characteristics were improved.

### 3.5. Sensory Evaluations of Highland Barley

**Figure 5:**
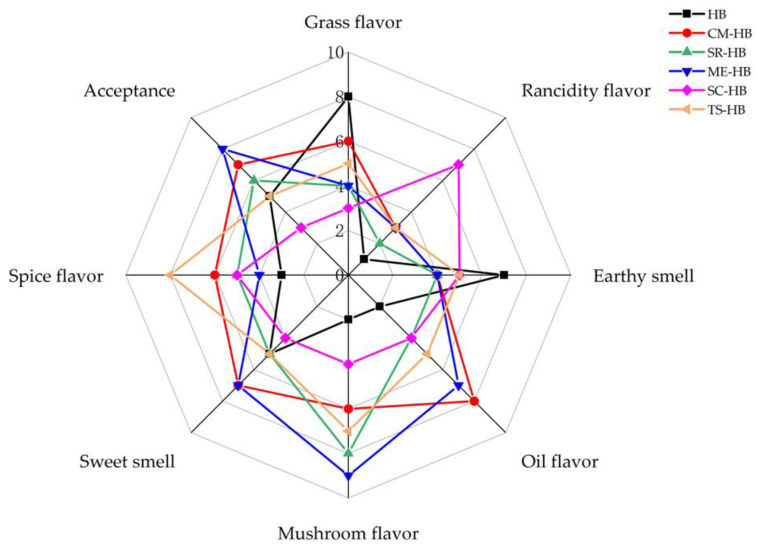
Flavor characteristics of original and fermented highland barley samples: HB (original highland barley), CM-HB (fermented highland barley with *C. militaris*), SR-HB (fermented highland barley with *S. rugoso-annulata*), ME-HB (fermented highland barley with *M. esculenta*), SC-HB (fermented highland barley with *S. commune*), TS-HB (fermented highland barley with *T. sanguinea*). 0 to 10 indicated the intensity of the different descriptors rated by each panelist in this study.

Sensory evaluations of the original and fermented highland barley were performed by direct sniffing and flavor-profile mapping. As shown in Figure 5, the original highland barley had a relatively strong grassy and earthy taste with moderate acceptance, but the flavor profiles were changed after fermentation. The flavor of highland barley was decreased after fermentation with *S. commune* and *T. sanguinea*, which was consistent with the results of ROAV. SC-HB showed a heavy sour and fermented odor with a low overall acceptability. TS-HB had a light overall odor with a strong spicy odor resulting in a lower acceptability of the sample. Fermentation with *C. militaris*, *S. rugoso-annulata* and *M. esculenta* showed significant improvement in sensory characteristics. Among them, the samples fermented with *M. esculenta* showed a significant sensory flavor improvement. This was in agreement with the results of GC-MS.

## 4. Conclusions

In this study, the flavor profiles of highland barley fermented with different mushroom mycelium were investigated. The results indicated that the strong grassy flavor of original highland barley was reduced due to the decease in hexanal, decanal and 2-pentylfuran after fermentation. The overall aroma of the fermented highland barley changed to mushroom, greasy and floral. Fermentation with mushrooms could improve the flavor of highland barley but depended on the fermented strains. *Cordyceps militaris*, *M. esculenta* and *S. rugoso-annulata* were able to reduce the undesirable odor of highland barley while producing a mushroom, oil and floral aroma. *Morchella esculenta* showed the best improvement and high acceptance for fermented highland barley. Additionally, the results can be used to develop new types of fermented products in the future.

## Figures and Tables

**Table 1:** Content of volatile compounds in original and fermented highland barley samples.

Number	RT	Unknown RI	Literature RI	Compounds	Aroma Characteristics	CAS#	Relative Content/(%)					
							HB	SC-HB	TS-HB	SR-HB	ME-HB	CM-HB
				**Aldehydes (16)**			61.67	52.83	38.49	44.68	36.88	45.17
1	26.34	1208	1206	Decanal	Green, Cucumber, Citrus	112-31-2	4.63 ± 0.27 ^a^	0.51 ± 0.10 ^d^	0.87 ± 0.07 ^cd^	1.32 ± 0.06 ^b^	0.97 ± 0.21 ^bc^	1.09 ± 0.12 ^bc^
2	6.86	802	800	Hexanal	Grass, Oil	66-25-1	33.34 ± 3.91 ^a^	0.59 ± 0.22 ^d^	8.66 ± 1.66 ^bc^	10.48 ± 1.98 ^b^	5.03 ± 0.89 ^c^	5.79 ± 1.25 ^c^
3	20.62	1109	1102	Nonanal	Fat, Fruity	124-19-6	6.23 ± 0.67 ^a^	1.08 ± 0.32 ^c^	1.17 ± 0.10 ^c^	1.79 ± 0.21 ^c^	4.12 ± 0.67 ^b^	1.15 ± 0.17 ^c^
4	10.74	901	902	Heptanal	Green, Fruity	111-71-7	3.89 ± 0.76 ^a^	ND	0.21 ± 0.07 ^b^	ND	0.21 ± 0.11 ^b^	0.39 ± 0.13 ^b^
5	18.07	1059	1049	Phenylacetaldehyde	Honey, Flower	122-78-1	4.67 ± 1.66 ^c^	13.82 ± 1.46 ^ab^	18.30 ± 2.01 ^a^	10.51 ± 0.54 ^b^	11.99 ± 1.53 ^b^	3.87 ± 0.23 ^c^
6	13.34	963	961	Benzaldehyde	Almond, Caramel	100-52-7	8.05 ± 0.95 ^b^	12.72 ± 1.30 ^a^	6.77 ± 0.42 ^c^	14.63 ± 1.11 ^a^	9.37 ± 0.68 ^b^	6.46 ± 0.30 ^bc^
7	18.64	1072	1064	trans-2-Octen-1-al	Grass, Oil	2548-87-0	0.86 ± 0.19 ^c^	0.25 ± 0.04 ^c^	1.17 ± 0.34 ^c^	2.55 ± 0.67 ^bc^	5.20 ± 1.33 ^b^	9.08 ± 1.36 ^a^
8	28.65	1278	1274	2-Phenylcrotonaldehyde	Musty, Floral, tea	4411-89-6	ND	1.45 ± 0.24 ^a^	ND	ND	ND	ND
9	11.38	917	911	3-methylthiopropionaldehyde	Stench	3268-49-3	ND	1.96 ± 0.31 ^a^	ND	ND	ND	ND
10	9.28	863	846	furan-3-carboxaldehyde	Burnt, Nutty aroma	498-60-2	ND	20.46 ± 2.41 ^a^	1.08 ± 0.07 ^b^	ND	ND	ND
11	33.75	1582	1573	Peach aldehyde	Peach	104-67-6	ND	ND	0.27 ± 0.05 ^a^	ND	ND	ND
12	30.49	1366	1362	gamma-nonanoic lactone	Fat, Coconut	104-61-0	ND	ND	ND	2.45 ± 0.38 ^a^	ND-	3.04 ± 0.86 ^a^
13	13.26	962	956	trans-2-Heptenal	Oil, Grass, Fruit	18829-55-5	ND	ND	ND	0.94 ± 0.12 ^a^	ND	ND
14	14.37	987	-	2-Ethyl-2-hexenal	-	645-62-5	ND	ND	ND	ND	ND	2.95 ± 0.45 ^a^
15	30.70	1378	1372	(E)-2-butyloct-2-enal	Green, Oily	13019-16-4	ND	ND	ND	ND	ND	5.88 ± 0.78 ^a^
16	12.75	949	-	Cyclohexanecarboxaldehyde	-	2043-61-0	ND	ND	ND	ND	ND	5.47 ± 1.20 ^a^
				**Alcohols (8)**	-		6.34	12.52	25.55	46.35	41.16	22.04
1	14.24	984	986	1-Octen-3-ol	Mushroom, Green	3391-86-4	6.34 ± 1.65 ^e^	9.31 ± 1.84 ^de^	15.42 ± 1.41 ^cd^	31.15 ± 2.68 ^b^	27.54 ± 0.79 ^a^	12.17 ± 1.24 ^c^
2	5.73	752	768	1-Pentanol	Mixed alcohol oil	71-41-0	ND	0.34 ± 0.07 ^a^	ND	ND	ND	ND
3	21.04	1116	1114	2-Phenylethanol	Wood incense	60-12-8	ND	2.40 ± 0.28 ^b^	7.10 ± 0.15 ^b^	ND	13.62 ± 1.45 ^a^	4.72 ± 0.35 ^b^
4	29.71	1319	1313	(1S,2S,3R,5S)-(+)-2,3-Pinanediol	Balsamic	18680-27-8	ND	0.47 ± 0.11 ^a^	ND	ND	ND	ND
5	18.82	1174	-	Cyclooctanol	-	696-71-9	ND	ND	1.89 ± 0.18 ^a^	ND	ND	ND
6	9.59	871	867	1-Hexanol	Grass fragrance	111-27-3	ND	ND	0.99 ± 0.17 ^b^	14.82 ± 1.32 ^a^	ND	1.11 ± 0.21 ^b^
7	20.62	1107	1104	Linalool	Floral, Lily of the valley	78-70-6	ND	ND	0.15 ± 0.02 ^b^	ND	ND	3.07 ± 0.28 ^a^
8	33.80	1584	1571	Nerolidol	Green, Floral, Fruity aromas	7212-44-4	ND	ND	ND	0.38 ± 0.04 ^b^	ND	0.97 ± 0.18 ^a^
				**Esters (7)**			4.71	1.94	29.59	0.66	1.81	19.32
1	20.50	1107	1091	Methyl benzoate	Honey, Flower	93-58-3	1.67 ± 0.32 ^b^	0.27 ± 0.08 ^c^	5.27 ± 0.83 ^a^	0.57 ± 0.10 ^bc^	0.56 ± 0.05 ^bc^	ND
2	31.05	1398	1378	Methyl 4-methoxybenzoate	Peppers, Herbs, Dried fruits	121-98-2	1.40 ± 0.15 ^b^	1.47 ± 0.32 ^b^	21.11 ± 1.85 ^a^	ND	ND	ND
3	26.73	1220	-	2-Ethylhexyl acrylate	-	103-11-7	1.63 ± 0.10 ^a^	ND	ND	ND	ND	ND
4	38.47	1975	1993	Ethyl palmitate	Fragrance, Milk	628-97-7	ND	0.20 ± 0.03 ^bc^	ND	0.09 ± 0.03 ^c^	0.56 ± 0.10 ^b^	3.50 ± 0.67 ^a^
5	3.23	596	605	Ethyl acetate	Pineapple, Apple	141-78-6	ND	ND	0.57 ± 0.06 ^a^	ND	ND	ND
6	40.31	2155	2159	Ethyl linoleate	Fatty	544-35-4	ND	ND	2.64 ± 0.16 ^b^	ND	0.69 ± 0.06 ^c^	10.61 ± 1.18 ^a^
7	40.22	2142	2149	Ethyl oleate	Cocoa	111-62-6	ND	ND	ND	ND	ND	4.54 ± 0.86 ^a^
8	36.11	1790	1793	Ethyl myristate	Iris aroma, Fat	124-06-1	ND	ND	ND	ND	ND	0.35 ± 0.07 ^a^
9	39.89	2104	2110	Ethyl stearate	Slightly waxy, Irritating	111-61-5	ND	ND	ND	ND	ND	0.32 ± 0.03 ^a^
				**Ketones (9)**			15.06	0.29	1.14	5.76	0	3.76
1	10.48	894	889	2-Heptanone	Banana, Medicine	110-43-0	3.35 ± 0.27 ^a^	ND	ND	1.42 ± 0.47 ^b^	ND	ND
2	31.81	1449	1458	(E)-6,10-Dimethyl-5,9-undecadien-2-one	Green, Magnolia, Fruit	3796-70-1	ND	0.29 ± 0.02 ^b^	1.14 ± 0.11 ^a^	ND	ND	ND
3	14.49	990	984	3-Octanone	Mushroom, Mould	106-68-3	ND	ND	ND	3.30 ± 0.39 ^a^	ND	ND
4	29.66	1317	-	4’-Hydroxy-2’-Methylacetophenone	-	875-59-2	11.71 ± 0.82 ^a^	ND	ND	ND	ND	ND
5	29.13	1293	1291	2-Undecanone	Waxy, Fruity	112-12-9	ND	ND	ND	0.66 ± 0.08 ^b^	ND	1.32 ± 0.24 ^a^
6	17.71	1054	1037	3-Octen-2-one	Nut, Crushed bug	1669-44-9	ND	ND	ND	0.38 ± 0.02 ^a^	ND	ND
7	33.30	1551	-	massoia lactone	Coconut	54814-64-1	ND	ND	ND	ND	ND	2.44 ± 0.14 ^a^
				**Acids (5)**			0.00	1.18	4.55	0.38	18.99	9.22
1	38.47	1986	1975	Palmitic acid	Waxy	57-10-3	ND	1.18 ± 0.11 ^b^	4.55 ± 0.08 ^a^	ND	4.79 ± 0.46 ^a^	3.89 ± 0.32 ^a^
2	40.16	2134	2140	Linoleic acid	Faint fatty	60-33-3	ND	ND	ND	ND	8.39 ± 0.78 ^a^	3.30 ± 0.26 ^b^
3	25.87	1196	1191	Octanoic acid	Cheese, Sweat, Spicy	124-07-2	ND	ND	ND	0.38 ± 0.04 ^a^	ND	ND
4	40.44	2170	2180	Stearic acid	Putrid	57-11-4	ND	ND	ND	ND	1.65 ± 0.23 ^a^	ND
5	28.14	1263	1280	Nonanoic acid	Green, Fat	112-05-0	ND	ND	ND	ND	4.27 ± 0.64 ^a^	3.03 ± 0.07 ^a^
				**Others (13)**			12.26	31.24	0.66	2.16	1.16	0.46
1	14.81	996	987	2-pentylfuran	Bean, Fruit	3777-69-3	7.86 ± 0.42 ^a^	0.71 ± 0.11 ^b^	0.27 ± 0.05 ^b^	0.98 ± 0.16 ^b^	ND	ND
2	26.11	1201	1200	Dodecane	Gasoline	112-40-3	0.74 ± 0.11 ^a^	0.10 ± 0.02 ^b^	ND	ND	ND	ND
3	34.03	1605	1600	n-Hexadecane	-	544-76-3	1.09 ± 0.12 ^a^	ND	ND	ND	ND	ND
4	31.10	1408	1400	Tetradecane	Alkane	629-59-4	1.52 ± 0.34 ^a^	2.21 ± 0.28 ^a^	ND	ND	ND	ND
5	32.81	1519	1521-	2,4-Di-tert-butylphenol	Leather	96-76-4	ND	0.42 ± 0.10 ^b^	ND	ND	1.16 ± 0.39 ^a^	0.46 ± 0.11 ^b^
6	32.48	1505	1502	(+)-Cuparene	-	16982-00-6	ND	ND	0.39 ± 0.07 ^a^	ND	ND	ND
7	33.17	1542	-	3,5-di-tert-butylphenol	-	1138-52-9	ND	0.34 ± 0.06 ^a^	ND	ND	ND	ND
8	26.52	1213	-	2,6-Dichloroanisole	-	1984-65-2	ND	ND	ND	0.28 ± 0.03 ^a^	ND	ND
9	29.68	1319	-	4-Ethyl-3-nonen-5-yne	-	74685-67-9	ND	ND	ND	0.56 ± 0.09 ^a^	ND	ND
10	32.29	1482	1483	α-curcumene	Herb	644-30-4	1.05 ± 0.06 ^a^	ND	ND	ND	ND	ND
11	29.99	1337	-	Phenol, 5-ethenyl-2-methoxy-	-	621-58-9	ND	27.47 ± 2.86 ^a^	ND	ND	ND	ND
12	29.35	1305	1315	2-Methylnaphthalene	Floral	91-57-6	ND	ND	ND	0.19 ± 0.02 ^a^	ND	ND
13	3.95	630	-	Ammonium acetate	-	631-61-8	ND	ND	ND	0.16 ± 0.03 ^a^	ND	ND

Note: “RT”: Retention time; “RI”: Retention Index; “ND”: volatile compounds not detected. Volatile compounds were identified by comparing the RI and MS fragmentation patterns with mass spectra from the NIST17 library. Different superscript lowercase letters in a row (a–d) represent statistically significant differences between the mean values at *p* < 0.05 determined by one-way ANOVA. CAS# is an alias of CAS number which indicates the unique numerical identification number of a substance.

**Table 2:** Comparison of the classification of volatile components in raw and fermented highland barley samples.

Compounds	Sample Categories					
	HB	SC-HB	TS-HB	SR-HB	ME-HB	CM-HB
Aldehydes	7	9	9	8	7	11
Alcohols	1	4	5	3	2	5
Esters	3	3	4	2	3	5
Ketones	2	1	1	4	0	2
Acids	0	1	1	1	4	3
Others	5	6	2	5	1	1
Total	18	24	22	23	17	27

**Table 3:** ROAV of volatile compounds in original and fermented highland barley samples.

Number	Compounds	Aroma Characteristics	Threshold μg/kg	ROAV					
				HB	SC-HB	TS-HB	SR-HB	ME-HB	CM-HB
1	Decanal	Green, Cucumber, Citrus	0.1	100	55.26	56.31	42.42	35.10	90.00
2	Hexanal	Grass, Oil	4.5	100	1.40	12.47	7.47	4.06	10.58
3	Nonanal	Fat, Fruity	1	84.01	11.58	7.57	5.76	14.98	9.43
4	Heptanal	Green, Fruity	2	26.25	ND	0.68	ND	0.39	1.60
5	Phenylacetaldehyde	Honey, Flower	4	15.75	37.11	29.66	8.43	10.58	7.95
6	Benzaldehyde	Almond, Caramel	300	0.36	0.46	<0.1	0.16	0.11	0.18
7	trans-2-Octen-1-al	Grass, Oil	3	3.85	0.88	2.52	2.73	6.29	24.87
8	3-(methylthio)propionaldehyde	Stench	0.2	ND	100	ND	ND	ND	ND
9	gamma-nonanoic lactone	Fat, Coconut	25	ND	ND	ND	0.32	ND-	1.72
10	trans-2-Heptenal	Oil, Grass, Fruit	13	ND	ND	ND	0.23	ND	ND
11	1-Octen-3-ol	Mushroom, Green	1	85.59	100	100	100	100	100
12	2-Phenylethanol	Wood incense	21	ND	1.23	2.19	ND	3.25	1.85
13	1-Hexanol	Grass fragrance	250	ND	ND	<0.1	0.19	ND	0.63
14	Linalool	Floral, Lily of the valley	6	ND	ND	0.16	ND	ND	4.21
15	Methyl benzoate	Honey, Flower	30	<0.1	<0.1	1.14	<0.1	<0.1	ND
16	Methyl 4-methoxybenzoate	Peppers, Herbs, Dried fruits	100	0.19	0.16	1.37	ND	ND	ND
17	Ethyl acetate	Pineapple, Apple	5	ND	ND	0.74	ND	ND	ND
18	Ethyl myristate	Iris aroma, Fat	4000	ND	ND	ND	ND	ND	0.20
19	Ethyl stearate	Slightly waxy, Irritating	10000	ND	ND	ND	ND	ND	0.18
20	2-Heptanone	Banana, Medicine	140	0.32	ND	ND	<0.1	ND	ND
21	(E)-6,10-Dimethyl-5,9-undecadien-2-one	Green, Magnolia, Fruit	60	ND	<0.1	0.12	ND	ND	ND
22	3-Octanone	Mushroom, Mould	28	ND	ND	ND	0.38	ND	ND
23	2-Undecanone	Waxy, Fruity	7	ND	ND	ND	0.30	ND	1.56
24	2-pentylfuran	Bean, Fruit	6	17.68	1.27	0.29	0.52	ND	ND

Aroma characteristics were retrieved from Flavornet. Compounds with ROAV ≥ 0.1 are presented. 0.1 ≤ ROAV < 1: the compound contributed little to the odor. ROAV > 1: the compound was a key volatile compound. “ND”: Not identified.

## Data Availability

Data is contained within the article.
